# Role of Physiotherapy in Osgood-Schlatter’s Disease in Adolescent Volleyball Smasher: A Case Report

**DOI:** 10.7759/cureus.53534

**Published:** 2024-02-04

**Authors:** Vaishnavi R Waghe, Swapnil U Ramteke

**Affiliations:** 1 Sports Physiotherapy, Ravi Nair Physiotherapy College, Datta Meghe Institute of Higher Education and Research, Wardha, IND

**Keywords:** volleyball smasher, volleyball athletes, knee pain, treatment, case report, pain, rehabilitation, adolescents, physiotherapy, osgood-schlatter’s disease

## Abstract

A 14-year-old female presented to the sports physiotherapy with a diagnosis of Osgood-Schlatter's disease (OSD), a condition particularly prevalent in adolescents engaged in sports with repetitive knee motions, such as volleyball. This ailment commonly manifests at the tibia, directly beneath the patella, eliciting discomfort and inflammation. The chronic overuse injuries involve repetitive activities inducing fatigue in specific anatomical structures. Adequate recovery mechanisms allow for tissue adaptation, mitigating the risk of injury. In the absence of proper recovery, microtrauma ensues, instigating inflammation mediated by substances like histamines. The release of inflammatory cells and enzymes inflicts damage on local tissue, and prolonged stress contributes to degenerative changes, resulting in weakness, diminished flexibility, and chronic pain. These manifestations are intimately associated with OSD in chronic or recurrent instances. The primary symptom of OSD is knee pain, often of sufficient severity to induce limping. Patients report discomfort during activities such as kneeling, descending stairs, prolonged stationary positions, prolonged episodes of sitting with the knee rendered immobile, and engagement in sporting activities. This case study specifically underscores the efficacy of tailored physiotherapy in the management of OSD among adolescent volleyball players. The study's findings indicate that the patient successfully alleviated symptoms, facilitating recovery with improved outcomes. Furthermore, the physiotherapy regimen appears instrumental in enhancing the patient's functional mobility, as evidenced by the study's outcomes.

## Introduction

Knee pain stands out as a prominent manifestation of discomfort, affecting one in three adolescents [1]. While knee discomfort is often perceived as self-limiting with no enduring consequences, it is intricately linked to a diminished quality of life (QOL) and decreased engagement in physical activities [2]. Osgood-Schlatter's Disease (OSD) is notably prevalent in dynamic, youthful populations a characterized by traction apophysitis of the tibial tubercle [3].

Patellofemoral pain (PFP) afflicts approximately 6% to 7% of adolescents, whereas OSD impacts around 10%, displaying varying degrees of severity [4,5]. Nearly 40% of patients report persistent discomfort during long-term follow-up, potentially exacerbating knee pain and fostering the development of tendinosis, thereby influencing the approach to surgical interventions [6].

Primary risk factors encompass stature, weight, body mass index (BMI), reduced flexibility in both lower limbs, height of the internal longitudinal arch of the supporting foot (with an elevated risk associated with a greater arch height), diminished ankle dorsiflexion of at least 10°, tibial rotations (indicated by an increase in the condyle-malleolar angle and external rotation of the tibia), concurrent genu valgum, and a pronated foot [7,8]. Strategic management of training loads, including the modulation of intensity levels during physical activity, quantity, and its adjustment, early specialization, and the rectification of nutritional imbalances-such as addressing vitamin D deficiency-may potentially contribute to the development of OSD, particularly in regions characterized by limited exposure to sunlight [6].

The pathophysiological cascade of chronic overuse injuries initiates with repetitive activities inducing fatigue in a specific anatomical structure, such as a tendon or bone. Adequate recovery periods allow the affected tissue to adapt to imposed demands, enabling it to withstand subsequent loading without succumbing to injury. Conversely, insufficient recovery leads to continuous stress and the development of microtrauma. This microtrauma triggers the body's inflammatory response, releasing vasoactive substances (histamines, leukotaxin, necrosin), mobilizing inflammatory cells (macrophages, lymphocytes, plasma cells), and damaging enzymes. In chronic or recurrent cases, persistent stress results in degenerative changes, manifesting as weakness, reduced flexibility, and chronic pain, closely associated with OSD [9].

## Case presentation

Patient information

A 14-year-old girl engaged in volleyball for one and a half years came to the sports outpatient department with a complaint of pain in her left leg just below the knee. The patient was apparently alright 1 year back when she experienced stumbling while playing volleyball and developed pain and swelling on the upper part of the tibia, just below the knee in the left lower limb. The patient presented with an absence of past medical history but due to repeated training procedures, she had undergone gradual degenerative changes for which she went to a private hospital where medications were given for pain relief. The pain had subsided for the time being but again reoccurred and she was unable to jump, climb stairs, or run properly for which she came to the sports department where assessment/investigations were done. On the Visual Analogue Scale (VAS), the score of pain was 4.8/10 on flexion movement of the knee joint and 0/10 on rest. Pain was at the anterior side of the left tibia below the knee, the pain was gradual in onset, dull aching in nature, and was aggravated on activity and relieved by rest and medications. There were no diurnal variations.

Clinical findings

After obtaining consent from the patient, the examination was done. As per the information provided by the patient, she complained of pain, which had gradually aggravated over the last 1 year. The patient was examined in a lying position. On local examination, grade II tenderness (patient complained of pain and winced) was present on the left lower limb below the knee. The physical examination was done using manual muscle testing (MMT), which is given in Table 1, and range of motion (ROM), given in Table 2.

**Table 1:** Manual muscle testing on day 1 2: Full range of motion (ROM) with gravity eliminated, 3: Full ROM against gravity

Variable	Muscles	Values
Manual muscle testing: Knee	Flexors	2/5
	Extensors	3/5

**Table 2:** Range of motion on day 1 AROM: Active range of motion, PROM: Passive range of motion

Variable	Joint movement	AROM	PROM
Range of motion: Knee joint	Flexion	0^o^-98^o^	0^o^-105^o^
	Extension	98^o^-0^o^	105^o^-0^o^

Physiotherapy intervention

Table 3 summarizes the patient's treatment protocol. The patient received physiotherapy treatment for 6 weeks and the pre- and post-treatment outcomes were recorded.

**Table 3:** Sports rehabilitation protocol [10-16] ROM: Range of motion, IFT: Interferential therapy, Kg: Kilogram, Hz: Hertz

Goals	Interventions	Dosage	Interventions	Dosage
	Week 0-3		Week 3-6	
Pain relief	Cryotherapy, IFT at low frequency (0-250 Hz) 4 pole vector, kinesio taping over the knee joint	Cryotherapy for 15-20 minutes, IFT for 10 minutes	Cryotherapy	15-20 minutes
Improve ROM	Bilateral heel slides	10 repetitions, 2 sets	hamstrings curls	10 repetitions, 2 sets
Improve flexibility	Regular static stretching of hamstrings, quadriceps, and calf muscles before and after exercise	5-10 mins	Regular static stretching of hamstrings, quadriceps, and calf muscles before and after exercise	10- 15 mins
Improve strength	Calf raises, squats, squats jump, dynamic quads with 1 kg resistance, clamshell exercise with red theraband, fire-hydrant with red theraband; hip bridges, bilateral leg raises for core strengthening.	10 repetitions of 2 sets	Squat sideways with red theraband; plank, crunches, bicycle crunches, and flutter kicks, wall sits with heels raised for core strengthening	10 repetitions of 2 sets, wall sits for 30 seconds 3 sets
Improve balance	Bilateral single-leg stance	30 seconds, 3 sets	Balancing on wobble board, lunges on trampoline for proprioception, dual task	30 seconds, 5 sets
Functional activities	Stairs climbing, walking	10-15 minutes	Inclined walking on treadmill, cycling and reverse cycling, swimming	10-15 minutes

**Figure 1:**
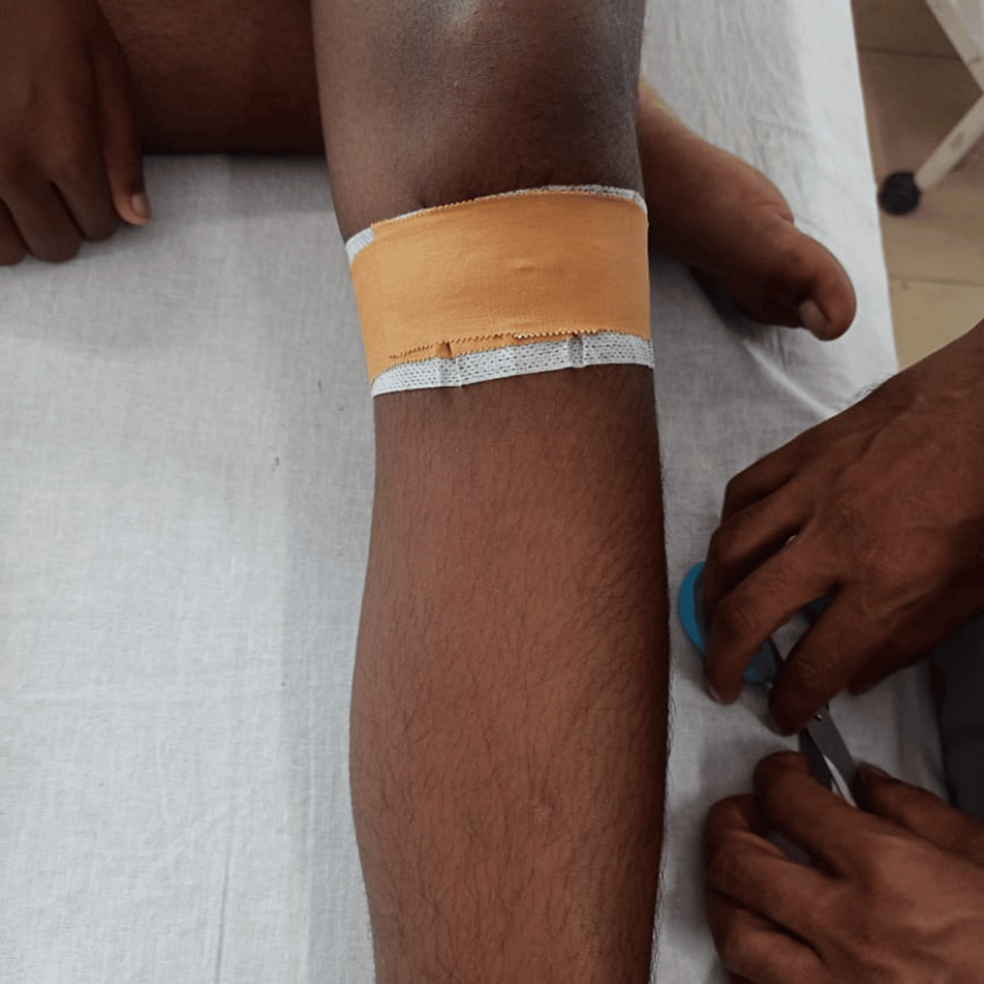
Taping for Osgood-Schlatter's disease

**Figure 2:**
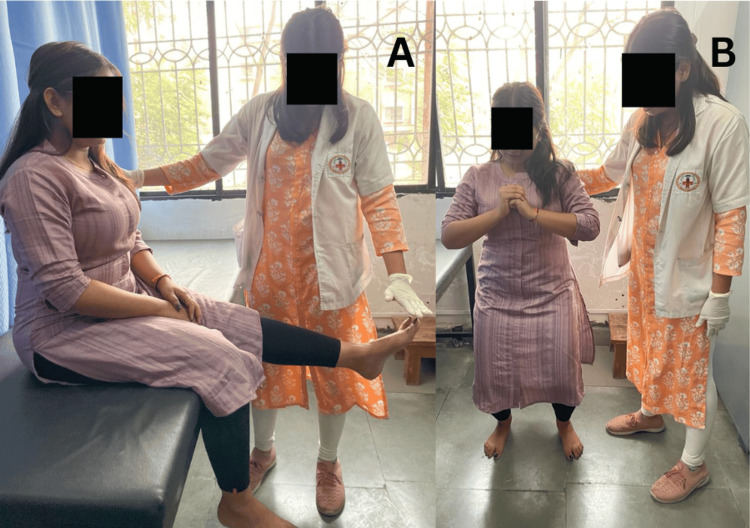
Patient performing (A) dynamic quadriceps and (B) semi-squats

Figure 1 shows the taping technique for OSD and Figure 2 shows the patient performing physical therapy. After six weeks of rehabilitation focused on core strengthening and eccentric control training, combined with taping, stretching, and functional activity training, significant improvements were observed. These advancements played a pivotal role in facilitating a notable enhancement, leading to a successful return to sports characterized by increased endurance and reduced awkward movements at the knee and ankle joints in further sports tournaments.

Follow-up and outcome measures

Tables 4, 5 show findings of outcome measures after 4 weeks of rehabilitation protocol, after which MMT and ROM were assessed.

**Table 4:** Manual muscle testing after physiotherapy treatment 5: Full range of motion against gravity with maximum resistance

Variable	Muscles	Values
Manual muscle testing: Knee	Flexors	5/5
	Extensors	5/5

**Table 5:** Range of motion after physiotherapy treatment AROM: Active range of motion, PROM: Passive range of motion

Variable	Joint movement	AROM	PROM
Range of motion: Knee	Flexion	0^o^-110^o^	0^o^-118^o^
	Extension	110^o^-0^o^	118^o^-0^o^

## Discussion

This study underscores the role of physiotherapy in the management of OSD among adolescent athletes, particularly those actively participating in high-impact sports like volleyball and basketball. In this specific case, the therapeutic approach adopts proximal core strengthening in conjunction with eccentric control training of the quadriceps. This intervention aims to optimize muscle control during landing, thereby attenuating the transmission of force to the patellar tendon and concurrently reducing overpull on the knee's extensor mechanism.

Additionally, taping is meticulously applied to the left lower limb below the knee, precisely where the patellar tendon attaches to the tibial shin. This taping serves a dual purpose: furnishing support and alleviating pressure at the knee joint. Successful outcomes indicate a comprehensive strategy addressing activity reduction, discomfort alleviation, strength enhancement, flexibility improvement, and biomechanical considerations.

OSD arises from recurrent stress on the quadriceps muscle and is more prevalent among young athletes, especially those initiating athletic training early in life [16]. Teenage athletes, owing to their underdeveloped bone structures, are particularly susceptible to this condition, with symptoms typically emerging between the ages of 8 and 13 for girls and 10 to 15 for boys during the rapid growth phase of adolescence. OSD is more frequently observed in males, particularly in those engaged in running and leaping sports [17].

The primary symptom of OSD is knee pain, often localized in the tibial shin below the knee, with or without concomitant swelling. The pain may manifest unilaterally or bilaterally, resulting in significant discomfort leading to limping. Affected individuals report pain during activities such as kneeling, descending stairs, prolonged stationary positions, prolonged sitting with the knee immobilized, and sports participation [18]. In those diagnosed with OSD, mechanical stress on the extensor mechanism, transmitted through the patellar tendon, disproportionately burdens the anterior aspect of the tibia, contributing to asymmetric growth patterns and an elevated posterior tibial slope [19]. OSD, being a self-limiting condition, typically resolves with skeletal maturity. Treatment is predominantly symptomatic, but persistent symptoms in adults unresponsive to conservative measures may necessitate surgical intervention, encompassing open, mesoscopic, and arthroscopic techniques [16].

Cryotherapy is employed for pain reduction and faster relief, and we have obtained similar results from our findings [20]. A study conducted by Antich and Brewster has concluded that patients with OSD exhibit notable improvement through physiotherapeutic intervention, encompassing a 5-minute ice massage to the tubercle area, quadriceps strengthening, straight leg raising and short arc quadriceps exercises [21]. Interferential therapy, enhancing blood circulation and expediting healing, is employed to alleviate pain by modulating various cell membrane currents based on the treated tissue. Specific frequencies within the range activate distinct physiological systems, expediting the healing process [22]. Utilizing preventative tape alongside traditional conditioning techniques may present a viable approach to delaying the onset of OSD [23].

## Conclusions

The case study underscores the pivotal role of physiotherapy as a primary intervention in the management of OSD in an adolescent volleyball player. The implemented physiotherapeutic approach, encompassing core strengthening with eccentric control training, and taping alongside stretching and functional activities training, proves instrumental in facilitating substantial improvements. This comprehensive intervention not only contributes to enhanced game performance but also fosters improved functionality and a quicker, safer return to sports for the patient within a significantly abbreviated timeframe.
